# Induction of 1-FEH in Mature Chicory Roots Appears to be Related to Low Temperatures Rather than to Leaf Damage

**DOI:** 10.1100/tsw.2002.857

**Published:** 2002-06-26

**Authors:** W. Van den Ende, A. Van Laere

**Affiliations:** Department of Biology, Laboratory for Developmental Biology, Botany Institute, KULeuven,Kasteelpark Arenberg 31, B-3001 Leuven, Belgium

**Keywords:** 1-FEH, 1-FFT, 1-SST, chicory (Cichorium intybus L.), fructan, inulin

## Abstract

Large-scale inulin production from chicory roots (Cichorium intybus L.) is hampered by the induction of 1-FEH activity (fructan 1-exohydrolase) and concomitant fructose production in autumn, coincident with a period with low night temperatures that cause leaf damage. To understand whether leaf damage per se is sufficient for 1-FEH induction and fructan breakdown, we defoliated mature chicory plants at a preharvest stage (September 10) and investigated the changes in carbohydrate levels and 1-FEH activities. Also, the activities of 1-SST (sucrose:sucrose 1-fructosyl transferase, EC 2.4.1.99), 1-FFT (fructan:fructan 1-fructosyl transferase, EC 2.4.1.100), and acid invertase (EC 3.2.1.26) were determined. Defoliation did not result in a prompt fructan breakdown and increase in 1-FEH activity, but after 10 days fructan breakdown and 1-FEH activities became higher in the defoliated plants. Defoliation resulted in a sharp decrease in 1-SST activity over the first 24 h. Afterwards, root 1-SST activities of defoliated plants remained at a lower level than in control plants. 1-FFT and invertase activities were not affected by defoliation. It can be concluded that defoliation of plants at the preharvest stage by itself did not induce the same rapid changes as observed in naturally induced October roots by low temperature (harvest stage). Taken together with our finding that 1-FEH is not induced in chicory roots when plants are transferred to the greenhouse early autumn (minimal temperature 14°C), we conclude that low temperatures might be essential for 1-FEH induction.

### INTRODUCTION

Fructans are the main reserve carbohydrate in the highly evolved and economically important plant families Poaceae, Liliaceae, and Asteraceae[1]. Their specific role remains obscure, but evolutionary and other data suggest a correlation with drought resistance or frost tolerance[2,3,4]. 1-SST (EC 2.4.1.99; G-F + G-F $\rightarrow$ G-F-F + G) and 1-FFT (EC 2.4.1.100; $G\text{-}(F)_n + G\text{-}(F)_m \Leftrightarrow G\text{-}(F)_{n-1} + G\text{-}(F)_{m+1}$ with n > 1, m > 0) are responsible for inulin-type fructan synthesis[5,6,7,8]. 1-FFT and 1-FEH ($G\text{-}(F)_n + H_2O \Rightarrow G\text{-}(F)_{n-1} + F$ with n > 1) catalyse the shift from high DP (degree of polymerisation) to low DP fructan afterwards[9,10,11]. For a long time, all enzymes and fructans were believed to be vacuolar[12]. However, the exclusive vacuolar localisation of fructan was questioned since the presence of fructan and FEH were reported in the apoplastic fluid[4].

During the last decade, industrial fructan production has increased from 1,000 to more than 100,000 tons annually. The majority of fructan is produced by food industries although important future industrial applications for fructan and fructan derivatives are also to be expected in nonfood industries for the production of adhesives, coatings, and cosmetics[13]. In northwestern Europe, the only useful crop for inulin production is chicory (*Cichorium intybus* L.) with yields comparable to sugar beet[14]. Chicory also is a promising crop for inulin production in the U.S.[15]. Large-scale inulin production from chicory however is hampered by the induction of 1-FEH activity and concomitant Fru production in autumn[11,16], necessitating an early harvest and quick processing. Moreover, the resulting high fructose concentrations together with high 1-FFT activity invariably lead to the production of reducing low DP fructans which lack a terminal glucose ($F_n$ series)[17] and participate in the Maillard reaction with amino acids during processing.

Effects of defoliation on fructan metabolism are well described in grasses that are extremely well adapted to grazing or cutting. The effect of cutting height on the carbohydrate metabolism in the haplocorm of timothy (*Phleum pratense* L.) was investigated[18]. These authors reported an induction of FEH in the haplocorm when the aerial parts were cut during the growing season, suggesting that the carbohydrates needed for leaf regrowth are supplied by fructan hydrolysis in the haplocorm. After defoliation of ryegrass, regrowth involved two different phases[19]. During the first phase, hexose (Hex), sucrose (Suc), and fructan levels decreased while the activity of FEH increased, the 1-SST activity decreased, and high acid invertase activities were found. During the second period of leaf regrowth FEH activity declined, 1-SST activity increased, and fructan synthesis resumed. We demonstrated that defoliation of chicory seedlings resulted in fructan breakdown by the induction of a specific 1-FEH (1-FEH II) in the roots[20]. So far no research has been done on the effect of defoliation on root fructan contents of mature field-grown chicory plants. However, 1-FEH activities were measured in rhizophores of *Vernonia herbacea* upon cutting of aerial organs[21]. FEH activity increased at about 4 days after sprouting of new shoots started.

Recent work suggested that the relative levels of activities of 1-SST, 1-FFT, and 1-FEH in yacon (*Polymnia sonchifolia*) could be involved in the determination of the maximal DP of the fructan molecules in this species[22]. A large increase in 1-FEH activity and a decrease in fructan concentration were also observed in the underground reserve organs of the yacon after strong typhoon winds caused physical damage to the aerial plant parts indicating that fructan is metabolized for the maintenance of growth when the supply of photosynthate is insufficient during the growing period[23].

Under natural conditions, chicory is a biennial plant, which is sown in May. During the first growing season, it produces a fleshy tap root containing inulin as a carbohydrate reserve. The tap root reaches near-maximal weight at the beginning of October. After overwintering as a rosette, root energy reserves are consumed for sprouting and flowering during the second growing season. For agronomical purposes (inulin extraction from commercial chicory; Belgian endive production from witloof chicory), mature tap roots are harvested at the end of September. Indeed, fructan breakdown by 1-FEH induction is usually observed in chicory around October 15, coincident with the first very low night temperatures that cause leaf damage. To understand whether leaf damage *per se* is sufficient for 1-FEH induction and fructan breakdown, we defoliated mature chicory plants at a preharvest stage (September 10) and then compared carbohydrate concentrations and 1-FEH enzymatic activities in defoliated and nondefoliated chicory roots. We also tested whether fructan depolymerisation and 1-FEH activity would increase in roots maintained under conditions which excluded leaf damage by low temperatures.

### MATERIALS AND METHODS

#### Plant Material, Growth Conditions, and Sampling

*C. intybus* L. var *foliosum* cv Flash was sown in rows on a field with sandy loam soil in Herent near Leuven (Belgium) on May 18. In the middle of this field, an area of about 3 m by 10 m (comprising 6 rows) was selected for sampling. Uneven rows (1, 3, 5) were defoliated (removal of about 90% of foliage) on September 10 and even rows (2, 4, 6) were not (control). Plants were sampled on September 10, 11, 12, 14, 16, 19, 22, 26 and on October 3, 10, 17, and 31. Each sampling date, we randomly took 3 roots per row. Tissues from 3 roots (from different rows) were randomly combined in one extract (3 extracts per condition).

Seeds from the same cultivar were also sown in plastic pots (diameter 25 cm, height 30 cm; one plant per pot) with perforated bottom, containing 10 l of loamy soil on May 26. Plants were grown to maturity in open air (September 18). At this time, half of the plants were transferred to the greenhouse in which a minimal temperature of 14°C was ensured. The other half remained outside. Half of the greenhouse plants received 16 h of light (PAR 350 $\mu$mol $m^{-2}$ $s^{-1}$). The other half received natural daylight.

Roots and leaves were rinsed with cold tap water, dried with paper towel, then leaf and root fresh mass (fm) were measured. Roots were subsequently peeled and cut radially to obtain a 10-g sample over the whole length of the root.

For the greenhouse experiment, samples for carbohydrate analysis and 1-FEH activity were taken throughout the period October 6 until December 1.

#### Extraction

Root tissue (30 g) was homogenized in a Waring-blender in 30 ml of ice-cold 50 mM Na-acetate buffer pH 5 containing 1 mM phenylmethylsulfonylfluoride, 10 mM $NaHSO_3$, 1 mM mercaptoethanol, 10 mM mannitol (as internal standard), 0.2 g $l^{-1}$ Na-azide, and 1 g $l^{-1}$ Polyclar AT (Serva, Heidelberg, Germany).

#### Carbohydrate Analysis and Protein Extraction

About 500 mg of the homogenate was diluted twofold with 0.2 g $l^{-1}$ Na-azide and incubated in a water bath at 95°C for 15 min. Further processing and carbohydrate analysis by HPLC (Dionex, Sunnyvale CA) were as earlier[24].

The homogenate was centrifuged for 5 min at 10,000*g*. Protein extract was obtained as described[24]. This extract was used for determining the protein concentration and the enzymatic activities of 1-SST, invertase, 1-FFT, and 1-FEH.

### Assay of 1-SST, Invertase, 1-FFT, and 1-FEH

Aliquots of the desalted protein extract were incubated for 2 h at 30°C (1-SST, invertase, 1-FEH) in 50 mM Na-acetate buffer pH 5 with 0.2 g $l^{-1}$ Na-azide containing either 50 mM Suc (1-SST, invertase) or 30 g $l^{-1}$ commercial chicory root inulin (DP ≥ 10, Sigma, St. Louis, MO; 1-FEH). For 1-FFT, aliquots of the protein extract were incubated for 2 h at 0°C in 100 mM MES-NaOH buffer pH 6.25 containing 30 g $l^{-1}$ commercial chicory root inulin (DP ≥ 10, Sigma) as a fructosyl donor, 10 mM Suc as a fructosyl acceptor, and 0.2 g $l^{-1}$ Na-azide. Reactions were stopped by heating at 95°C for 5 min. Carbohydrates were subsequently analysed[24]. Enzyme activities are expressed in units (U) $g^{-1}$ protein. Protein contents were assayed as earlier[24]. One unit activity is defined as the amount of enzyme, which produced 1 µmol of 1-Kes from Suc (1-SST), 1 µmol Fru from Suc (invertase), 1 µmol Fru from inulin (1-FEH), or 1 µmol 1-Kes from inulin and Suc (1-FFT) per minute.

For the greenhouse experiment, we focused on 1-FEH activity. We did not determine protein contents and activities were expressed in nmoles $g^{-1}$ fm $min^{-1}$.

### Statistics

Data points represent means of three samples (each containing material of three plants). The corresponding SE are indicated. A classical *t*-test was performed to demonstrate statistical difference between 1-FEH activities in control plants and defoliated plants.

## RESULTS

### Growth Characteristics after Defoliation of Field-Grown Chicory Plants

Throughout several growing seasons, we demonstrated that the root fm of chicory remains fairly constant during September and October[11]. In contrast, the leaf fm decreased during this period. Field-grown chicory plants (preharvest stage) were defoliated (removal of about 90% of the foliage) on September 10 whereby the shoot to root ratio decreased abruptly from 1.8 to 0.7. The ratio decreased even further thereafter (Fig. 1), due to a loss of leaf fm, as root fm was rather stable (data not shown). For the nondefoliated plants, the shoot to root ratio decreased abruptly between September 22 and October 17 (Fig. 1).

### Root Carbohydrate Concentrations after Defoliation of Field-Grown Chicory Plants

In the control plants little variation in carbohydrate concentrations was found between September 10 and October 17 (Fig. 2). Fructan depolymerisation was initiated between October 17 and October 31 apparently resulting in higher concentrations of Hex + Suc and lower concentrations of fructan with DP > 20. The same trend was observed in the defoliated plants but fructan depolymerisation started around September 20 resulting in higher Hex + Suc concentrations and lower high DP fructan concentrations (Fig. 2A, C). From October 10 on, the DP3-20 fructan concentration was higher than that of the control plants (Fig. 2B) and the Hex + Suc levels were twice as high as in the control plants (Fig. 2A).

For the defoliated plants, similar results were obtained when these three carbohydrate classes were expressed as a percentage of total carbohydrate (“relative concentration”). Hex + Suc and

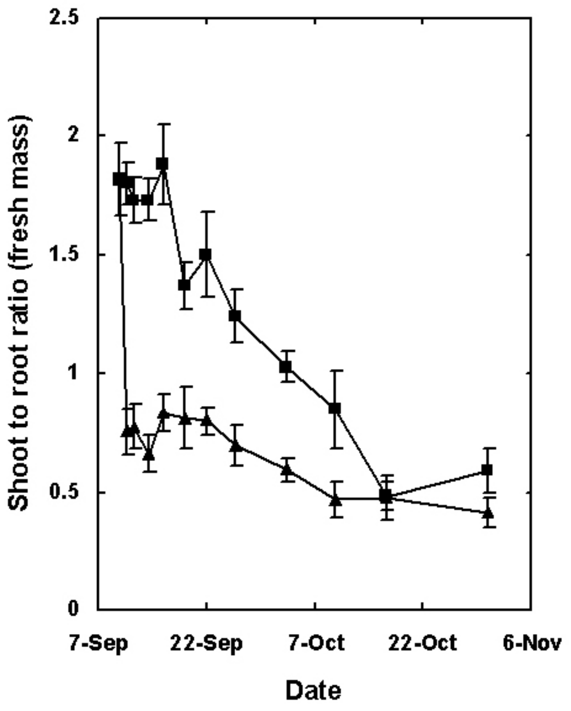

**FIGURE 1**. Changes of the shoot to root ratio (g fm) in control (■) and defoliated (▲) field-grown chicory plants.

high DP fructan (DP > 20) concentrations became higher and lower, respectively while the DP3-20 relative fructan concentration remained the same (data not shown). Starting from the end of September, both the absolute as well as the relative concentrations of fructan lacking terminal glucose ($F_n$ series containing the inulo-*n*-ose series)[17,25] were much higher in the roots of the defoliated plants than in the control plants (Fig. 3).

### Changes of Root Enzymatic Activities after Defoliation of Field-Grown Chicory Plants

During the first 24 h after defoliation, 1-SST activity decreased sharply (Fig. 4A) and remained much lower than in the control throughout September. Interestingly, this abrupt 1-SST decrease was not accompanied by a prompt 1-FEH increase (Fig. 4C). In the defoliated plants, no obvious changes were observed in 1-FFT or invertase activity as a function of time and no difference could be detected between intact and defoliated plants (Fig. 4B,D). The activity of 1-FEH fluctuated throughout the period under investigation, but the activity on October 31 was considerably higher than on September 10. From about 10 days after defoliation, the 1-FEH activity became higher in the defoliated plants (Fig. 4C; *t*-test: $p < 0.05$).

### Carbohydrates and 1-FEH Activity Changes in Field-Grown and Greenhouse Plants

Fig. 5 clearly demonstrates the gradual increase in 1-FEH activity in chicory roots kept outside over the period early October to early December. As already explained in detail in previous

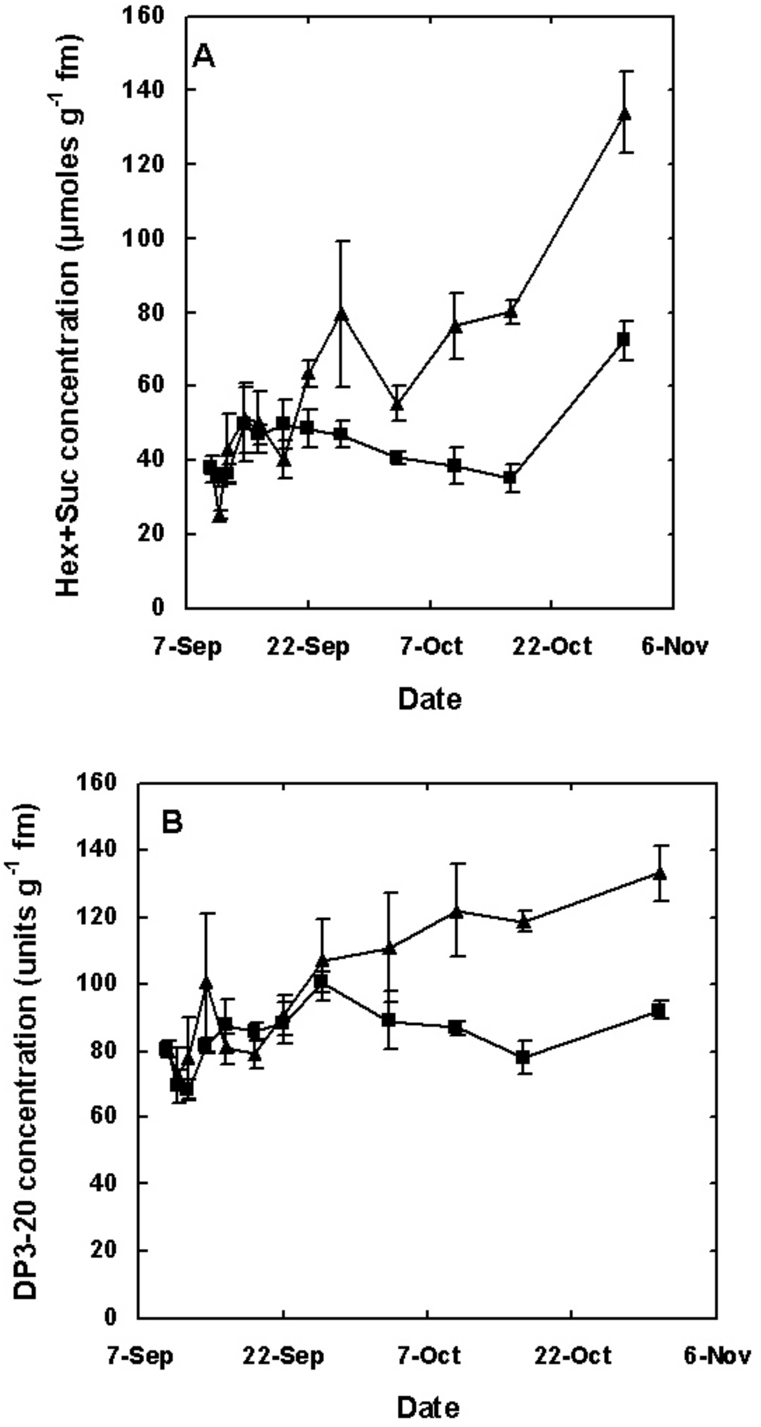

**FIGURE 2**. Changes of the Hex + Suc (A), DP3-20 fructan (B) and fructan with DP > 20 (C) concentrations in the roots of intact (■) and defoliated (▲) field-grown chicory plants.

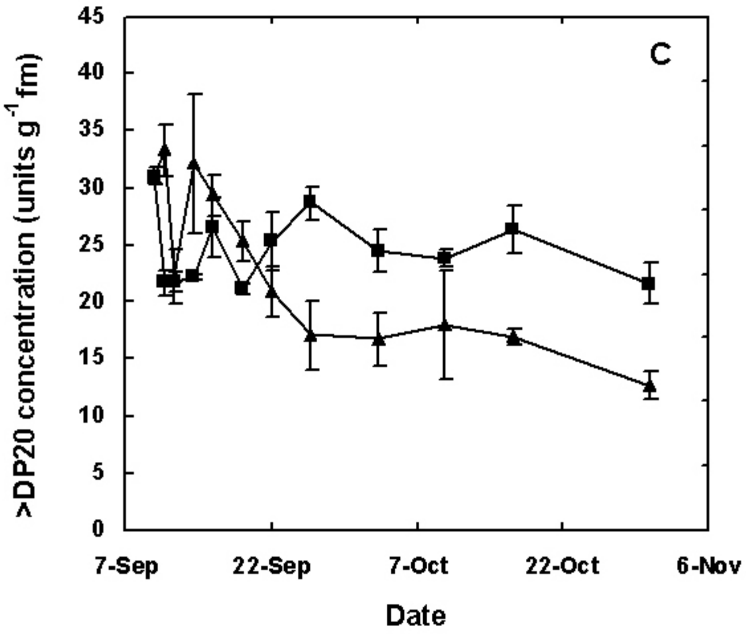

**FIGURE 2C.**

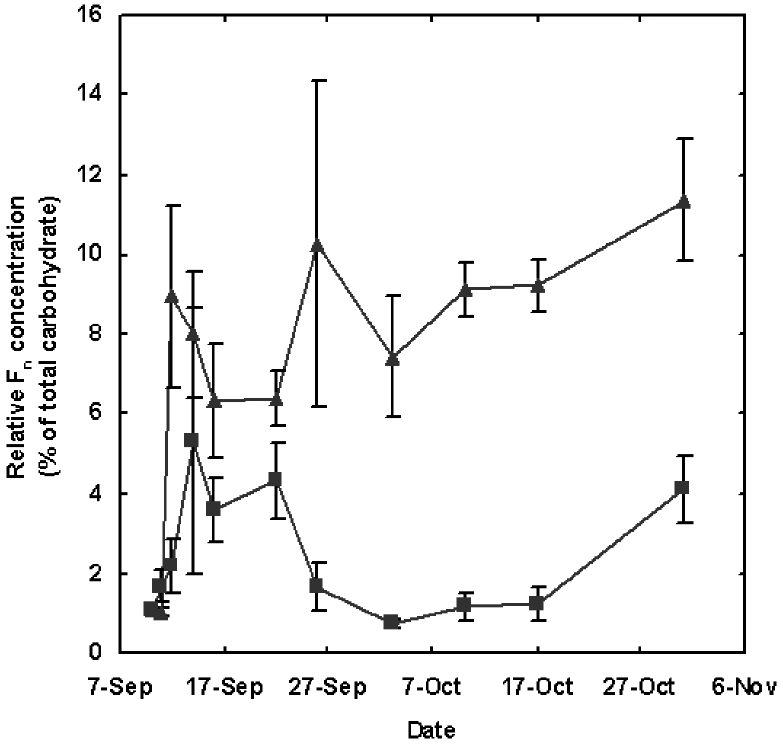

**FIGURE 3**. Changes in the “relative concentration” of fructan lacking terminal Glc ($F_n$ series) in the roots of intact (■) and defoliated (▲) field-grown chicory plants.

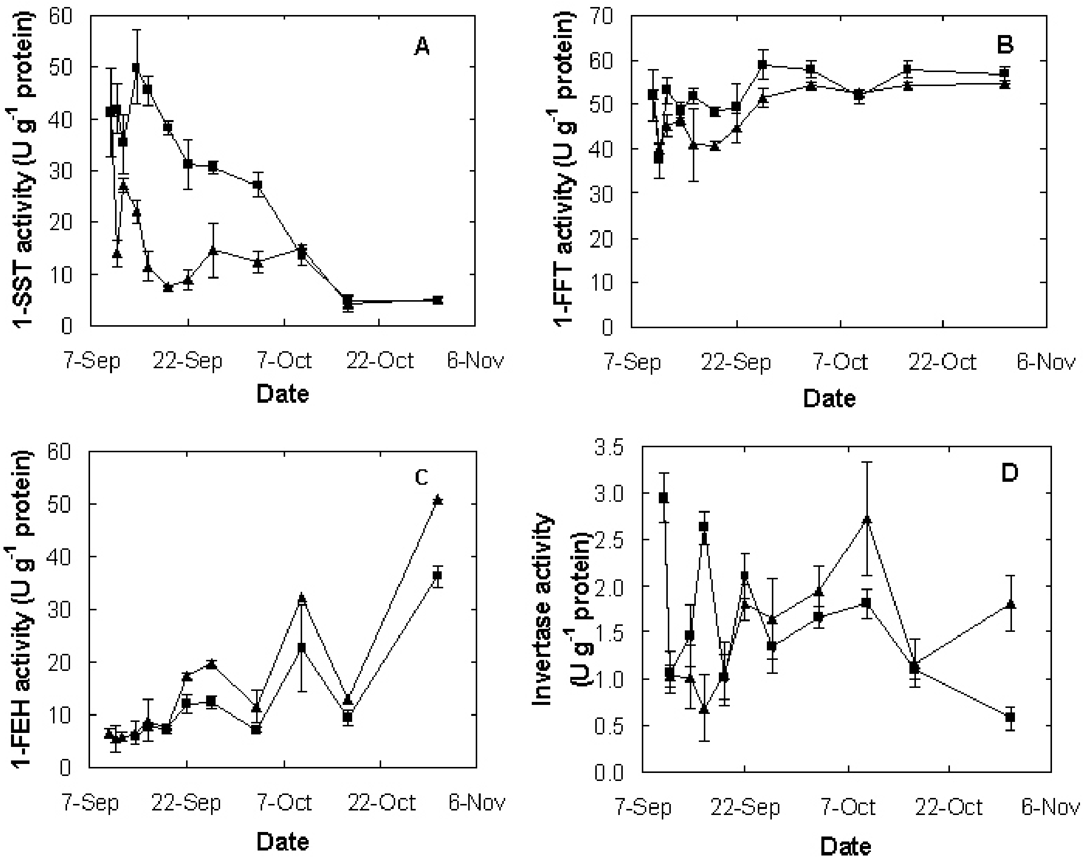

**FIGURE 4**. Changes of the activities of 1-SST (A), 1-FFT (B), 1-FEH (C), and acid invertase (D) in the roots of intact (■) and defoliated (▲) field-grown chicory plants.

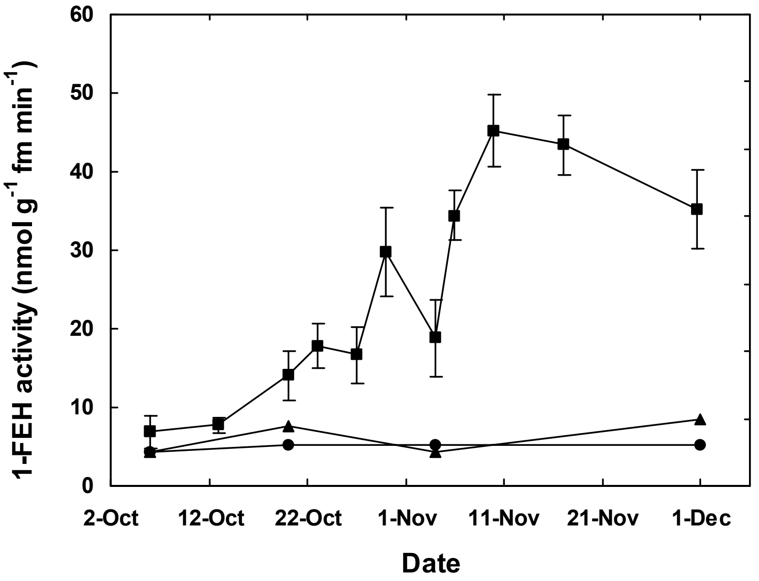

**FIGURE 5**. Changes in 1-FEH activity in autumn chicory roots. Outside plants (■) and greenhouse plants with (▲) or without (♦) additional light.

reports, these activity changes were well correlated with drastic changes in carbohydrate levels[11,16]. Over the same period, no 1-FEH activity increase (Fig. 5) and fructan depolymerisation (data not shown) was observed in the greenhouse plants.

### DISCUSSION

Fructan depolymerisation by induction of 1-FEH activity at the preharvest stage is a major problem for carbohydrate industries dealing with inulin extraction from chicory roots. Previous research on chicory indicated that the period of the increase of 1-FEH activity and fructan depolymerisation coincided with the first very cold nights with temperatures below 5°C[11]. It seems that FEH activity in plants can be induced at any time when energy demands highly increase, e.g., for leaf regrowth after switching young chicory plants from a N-deficient to a N-rich solution[26], for leaf regrowth after defoliation of grasses[18,19], or sprouting after cutting away the leaves of *V. herbacea*[21]. In these particular cases, it is clear that low temperature is not the essential trigger for induction of FEH gene induction. The Suc level in the cell might control fructan biosynthesis and breakdown[26,27]. Based on own former results, we also suggested that a sudden drop in Suc influx into particular cells might be the trigger for upregulating the 1-FEH II gene and downregulating the 1-SST gene[20,26].

To learn more about the putative 1-FEH regulation by the incoming Suc entering mature chicory roots, we defoliated plants in September (before the usual start of fructan depolymerisation). Similar to results obtained with grasses and chicory seedlings[19,20], defoliation resulted in a very rapid (after 1 day) decrease in root 1-SST activity (Fig. 4A), suggesting that the 1-SST gene is quickly downregulated when incoming root Suc supplies decrease. Similar results were also obtained in *V. herbacea* roots after defoliation[21]. When young chicory seedlings are defoliated, the fast 1-SST downregulation was also correlated with a prompt 1-FEH upregulation[28]. Interestingly, the latter is not observed in mature, field-grown chicory roots since no prompt 1-FEH induction (Fig. 4C) or fructan breakdown (Figs. 2 A and C) is observed during the first 10 days after defoliation. This might indicate that 1-FEH regulation might be different at different developmental stages or that different FEHs are involved. It can be concluded that the regulation of 1-FEH in field-grown chicory plants must be more complicated than that of 1-SST, and that other factors than Suc play a role.

The observed difference in chicory root 1-FEH response after defoliation between young and mature stages might be explained by the fact that in these mature chicory plants no energy supplies from the root are needed for a net regrowth of the photosynthetic apparatus. Indeed, in contrast to the results obtained on *V. herbacea*[21], no sprouting did occur and shoot to root ratios even decreased further after defoliation due to a loss in leaf fm (Fig. 1). Maybe this marked difference can be explained by the fact that the chicory defoliation was performed in early autumn, when temperatures start to decrease and days become shorter. These conditions are unfavourable for sprouting and very likely the sprouting process itself must be triggered before the onset of fructan depolymerisation[21]. Compared to chicory seedlings, mature chicory roots seem much more "buffered" against drastic changes in assimilate supply such as caused by defoliation because they already contain a very large amount of inulin.

Taken all together, the 1-FEH–activity induction in chicory roots later in autumn (after October 15) can probably not be explained by leaf damage and a lower influx of Suc into the root. It can be concluded that the removal of the photosynthetic apparatus from September plants is not *by itself* enough to induce the drastic and rapid changes usually observed in the October plants over a period with low temperatures. This result is important because we can now abandon our former hypothesis that leaf damage, *indirectly* caused by low temperatures, is sufficient for 1-FEH induction[11]. However, the susceptibility to 1-FEH induction is enhanced in defoliated plants from 10 days after defoliation (Fig. 2, Fig. 4C). As a new working hypothesis, we suggest that a *direct* trigger is needed for 1-FEH induction in autumn chicory roots. As triggers, both shortening of days and lower temperatures at the end of the growing season (or a combination of them) are plausible candidates. Since we were not able to induce the observed changes by shortening days from September 10 on[11], low temperature is the most plausible candidate as an essential primary trigger for 1-FEH induction in autumn chicory roots. Indirect evidence for this comes from the fact that no 1-FEH induction is observed when chicory plants are protected against cold by transferring them to the greenhouse in September (Fig. 5). This experiment on itself has practical implications for the commercial fructan industries. Indeed, the problems of FEH induction and decrease in fructan quantity and quality can most probably be circumvented by growing chicory at warmer climates (lower latitudes). From an energetic point of view, the reason for the drastic fructan depolymerisation in autumn chicory roots still remains obscure since at that time no energy is needed for growth. Interestingly, despite the fact that there is no prompt induction of 1-FEH activity (Fig. 4C) and accompanying fructan depolymerisation (Fig. 2) after defoliation, apparently more $F_n$ - type fructans are generated after a while, especially after the end of September (Fig. 3). However, this increase in $F_n$ can probably not be explained by an increase in 1-FFT activity (Fig. 4B). It cannot be ruled out that Hex, Suc, and low DP fructan (including $F_n$ type oligofructans) in overwintering roots may have a function as cryo-protectants[4]. If fructans indeed are present in the apoplast, they could protect the cell membrane against drought (e.g., by replacing water for membrane stabilisation) or frost (freezing point decrease by high concentration of fructans in a small apoplastic volume surrounding the cells). Recently, fructans have been shown to protect membranes during freeze-drying[29]. It was further convincingly demonstrated that the conversion of fructan to cryoprotective sugars in response to low temperatures, especially subzero ones, is different in wheat cultivars varying in freezing tolerance[30].

### CONCLUSION

Unexpectedly, defoliation of September chicory roots resulted in an abrupt decrease in 1-SST activity but, in contrast to chicory seedlings, this was not accompanied by a prompt increase in 1-FEH activity and fructan depolymerisation. However, from 10 days after defoliation, 1-FEH activity and fructan breakdown was higher in the defoliated plants compared to the control plants. Transfer of chicory to the greenhouse prevents 1-FEH induction and thus growing chicory at warmer climates would circumvent the problem of 1-FEH induction as observed in autumn-derived chicory roots in Western Europe.

### ACKNOWLEDGEMENTS

The authors want to thank E. Nackaerts for his technical assistance. W. Van den Ende is supported by grants from the Fund for Scientific Research (FSR, Flanders).
